# Supplementary material for: Health-related quality of life and utility of maternity health states amongst post-partum Australians
Source: PLoS One. 2024 Oct 7;19(10):e0310913. doi: 10.1371/journal.pone.0310913 (PMC11457989; doi:10.1371/journal.pone.0310913)
Supplement: S1 Table — (DOCX) [file pone.0310913.s001.docx]

**Supplementary File 1.**

Health-related quality of life and utility of maternity health states amongst post-partum Australians

Authors

**S1 Table.** EQ-5D-3L prevalence and 95% confidence intervals by domain and level of impairment over the six-week postpartum period.

|  | 1-week  % (95% CI)  *n* = 125 | 2-weeks  % (95% CI)  *n* = 66 | 3-weeks  % (95% CI)  *n* = 56 | 4-weeks  % (95% CI)  *n* = 67 | 5-weeks  % (95% CI)  *n* = 55 | 6-weeks  % (95% CI)  *n* = 60 |
| --- | --- | --- | --- | --- | --- | --- |
| Mobility |  |  |  |  |  |  |
| No problems | 86.4 (79.3–91.3) | 97.0 (89.6–99.2) | 96.4 (87.9–99.0) | 100.0 (94.9–100.0) | 96.2 (87.3–99.0) | 98.3 (91.0–99.7) |
| Some problems | 12.8 (8.0–19.8) | 3.0 (0.8–10.4) | 3.6 (1.0–12.1) | – | – | 1.7 (0.3–9.0) |
| Severe problems | 0.8 (0.1–4.4) | – | – | – | 3.8 (1.0–12.8) | – |
| Personal Care |  |  |  |  |  |  |
| No problems | 98.4 (94.4–99.6) | 98.5 (92.0–99.7) | 98.2 (90.1–99.7) | 100.0 (94.6–100.0) | 100.0 (93.5–100.00) | 98.3 (91.1–99.7) |
| Some problems | 0.8 (0.1–4.4) | 1.5 (0.2–8.0) | 1.8 (0.3–9.5) | – | – | 1.7 (0.3–8.8) |
| Severe problems | 0.8 (0.1–4.4) | – | – | – | – | – |
| Usual Activities |  |  |  |  |  |  |
| No problems | 65.6 (56.9–73.4) | 83.6 (72.9–90.6) | 85.7 (74.3–92.6) | 92.2 (83.0–96.6) | 89.7 (79.2–95.2) | 95.0 (86.3–98.3) |
| Some problems | 28.0 (20.9–36.4) | 13.4 (7.2–23.6) | 14.3 (7.4–25.7) | 7.8 (3.4–17.0) | 6.9 (2.7–16.4) | 5.0 (1.7–13.7) |
| Severe problems | 6.4 (3.3–12.1) | 3.0 (0.8–10.3) | – | – | 3.4 (1.0–11.8) | – |
| Pain and Discomfort |  |  |  |  |  |  |
| No problems | 80.8 (73.0–86.7) | 93.9 (85.4–97.6) | 94.6 (85.4–98.2) | 95.6 (87.8–98.5) | 96.4 (87.7–99.0) | 95.0 (86.3–98.3) |
| Some problems | 16.8 (11.3–24.3) | 6.1 (2.4–14.6) | 3.6 (1.0–12.1) | 2.9 (0.8–10.1) | – | 5.0 (1.7–13.7) |
| Severe problems | 2.4 (0.8–6.8) | – | 1.8 (0.3–9.5) | 1.5 (0.3–7.9) | 3.6 (1.0–12.3) | – |
| Anxiety or Depression |  |  |  |  |  |  |
| No problems | 94.4 (88.9–97.3) | 92.5 (83.7–96.8) | 94.6 (85.4–98.2) | 95.6 (87.8–98.5) | 90.9 (80.4–96.1) | 91.7 (81.9–96.4) |
| Some problems | 4.8 (2.2–10.1) | 6.0 (2.4–14.4) | 5.4 (1.8–14.6) | 4.4 (1.5–12.2) | 7.3 (2.9–17.3) | 8.3 (3.6–18.1) |
| Severe problems | 0.8 (0.1–4.4) | 1.5 (0.3–8.0) | – | – | 1.8 (0.3–9.6) | – |

CI, Confidence Interval.

*Note:* Sample size varies by domain and time-period due to missing values.

– indicates cells with 0 responses for the category.
