# Supplementary material for: Health-related quality of life and utility of maternity health states amongst post-partum Australians
Source: PLoS One. 2024 Oct 7;19(10):e0310913. doi: 10.1371/journal.pone.0310913 (PMC11457989; doi:10.1371/journal.pone.0310913)
Supplement: S2 Table — (DOCX) [file pone.0310913.s002.docx]

**Supplementary File 2.**

Health-related quality of life and utility of maternity health states amongst post-partum Australians

Authors

**Comparison to Population Norms for Queensland, Australia**

Mean utility values at each week for the first six weeks following birth were significantly lower when compared Queensland, Australia population norms for females aged 25–34 years (Table S2). Mean utility values for women aged 25–34 years and women aged 35–44 years at one week postpartum were significantly lower than Queensland population norms for females in the same age brackets (Table S2). Mean utility values for 18–24 and 45–54-year age brackets for the study sample were not calculated due to low frequencies of participants in these categories.

**S2 Table.** Mean utility values over the six-week postpartum period and across age categories compared with Queensland, Australia population norms for females.

|  | Study Sample | Queensland Population Norms^a^ | Differences across value sets | |
| --- | --- | --- | --- | --- |
|  | Mean (95% CI) | Mean (95% CI) | *t* (*df*) | *p*-value |
| Time |  |  |  |  |
| 1 week postpartum (*n* = 125) | 0.71 (0.68–0.74) | 0.91 (0.89–0.93)^b^ | -15.057 (124) | <.001 |
| 2 weeks postpartum (*n* = 66) | 0.78 (0.75–0.81) | 0.91 (0.89–0.93)^b^ | -7.907 (65) | <.001 |
| 3 weeks postpartum (*n* = 56) | 0.81 (0.78–0.85) | 0.91 (0.89–0.93)^b^ | -4.870 (55) | <.001 |
| 4 weeks postpartum (*n* = 67) | 0.85 (0.82–0.88) | 0.91 (0.89–0.93)^b^ | -4.256 (66) | <.001 |
| 5 weeks postpartum (*n* = 55) | 0.85 (0.80–0.90) | 0.91 (0.89–0.93)^b^ | -2.577 (54) | .013 |
| 6 weeks postpartum (*n* = 60) | 0.87 (0.87–0.91) | 0.91 (0.89–0.93)^b^ | -2.293 (59) | .025 |
| Age^c^ |  |  |  |  |
| 18–24 years | – | 0.90 (0.88–0.93) |  | – |
| 25–34 years (*n* = 75) | 0.70 (0.67–0.74) | 0.91 (0.89–0.93) | -10.649 (74) | <.001 |
| 35–44 years (*n* = 46) | 0.72 (0.69–0.76) | 0.89 (0.87–0.90) | -9.854 (45) | <.001 |
| 45–54 years | – | 0.85 (0.84–0.87) |  | – |

CI, Confidence interval; df, Degrees of Freedom.

*Note:* Sample size varies between weeks postpartum due to missing values.

^a^ From Clemens, S., Begum, N., Harper, C., Whitty, J. A., Scuffham, P. A. (2014). A comparison of EQ-5D-3L population norms in Queensland, Australia, estimated using values sets from Australia, the UK and USA. *Quality of Life Research, 23*(8), 2375-2381.

^b^ Queensland, Australia population norm for females aged 25–34 years.

^c^ Utility value at one week postpartum
