# Supplementary material for: Health-related quality of life and utility of maternity health states amongst post-partum Australians
Source: PLoS One. 2024 Oct 7;19(10):e0310913. doi: 10.1371/journal.pone.0310913 (PMC11457989; doi:10.1371/journal.pone.0310913)
Supplement: Attachment — Submitted filename: Response to Reviewers.docx [file pone.0310913.s003.docx]

Dear Editor,

In this table, we have itemised the feedback we received and responded to each.

| **General requirements** | **Authors’ response** |
| --- | --- |
| 1. Please ensure that your manuscript meets PLOS ONE's style requirements, including those for file naming. | We have ensured this. |
| 2. Did you know that depositing data in a repository is associated with up to a 25% citation advantage (https://doi.org/10.1371/journal.pone.0230416)? If you’ve not already done so, consider depositing your raw data in a repository to ensure your work is read, appreciated and cited by the largest possible audience. | We have deposited the data in the University of Queensland’s data repository. [https://doi.org/10.48610/c933a47](https://doi.org/10.48610/c933a47) |
| 3. Thank you for stating the following in the Competing Interests section: "Authors EM and JT previously worked for the health service involved in this study but were not employed at the time of data collection. MB works for the health service involved in this study."  Please confirm that this does not alter your adherence to all PLOS ONE policies on sharing data and materials, by including the following statement: ""This does not alter our adherence to  PLOS ONE policies on sharing data and materials.” (as detailed online in our guide for authors [http://journals](http://journals/).plos.org/plosone/s/competing-interests).  If there are restrictions on sharing of data and/or materials, please state these.  Please include your updated Competing Interests statement in your cover letter; we will change the online submission form on your behalf. | We have included our updated Competing interest statement in our cover letter for this resubmission. |
| 4. In the online submission form, you indicated that "Due to the sensitive nature of the data, an anonymised version of the data can be requested from the corresponding author."  All PLOS journals now require all data underlying the findings described in their manuscript to be freely available to other researchers, either 1. In a public repository, 2. Within the manuscript itself, or 3. Uploaded as supplementary information. | Our data is now freely available to other researchers in the public repository: [https://doi.org/10.48610/c933a47](https://doi.org/10.48610/c933a47) |
| 5. Please include captions for your Supporting Information files at the end of your manuscript, and update any in-text citations to match accordingly. | We have included caption for our two Supporting Information files at the end of the manuscript. |
| **Reviewer one comments** |  |
| 1. My biggest concern to this study is that I did not see whether it was adequately representative and powered to detect the outcomes that the study intended. While they reported the inclusion and exclusion criteria, it is important to show the sample size calculation to justify that the 157 that consented were adequate to study these outcomes. | Thank you to the reviewer for pointing out this gap in our methods. As there is no existing research or data on which to base the sample size calculation e.g., for power or the effect of time on health-related quality of life, we followed the criteria suggested by Green to estimate an appropriate sample size. The following text has been added to the manuscript:  The required sample size for regression analysis was determined according to Green’s rule of thumb, where N(sample size) > 50+8m (independent variables) in the case of multiple correlations and N> 104+m in the case of testing individual predictors. Accordingly, the minimum number of participants needed for this study was 98 [N>50+(8×6)], and the minimum number of samples needed for testing individual predictors was 110. |
| 2. The exclusion of 19 participants with missing data is also of concern to me as it brings in selection bias. The authors may consider including these participants in the analysis and can consider doing multiple imputation as the percentage of 12.1% missing data can be imputed to avoid per protocol analysis in line with STROBE Guidelines. | The reviewer is correct that the exclusion of 19 participants with missing data may bring in selection bias. We do not think it is appropriate to use multiple imputations for the 15 participants who did not complete the survey, as this data may not be missing at random (e.g., the women were not well enough/completing a survey was difficult with a newborn). We also cannot make assumptions about their HRQoL through multiple imputations.  When considering the four remaining participants who were also excluded, then that is only an exclusion of 2.8% of the sample who completed at least 1 timepoint (n=138), which is why we used listwise exclusion rather than multiple imputation.  Regarding the avoidance of per protocol analysis to minimise bias as per the STROBE Guidelines, we have addressed this issue further in limitations. |
| 3. The differences in the sample population with the target population that gave birth at the three facilities by age group 18 – 24 but no differences when age group is stratified by below and above 35 years demonstrates that there was selection bias with the finer stratification of 18-24 which is masked when larger strata are used of below and above 35 years. I suggest you modify table 1 to reflect this and later acknowledge the sample deviation from the target population in the limitations. | Thank you for pointing this out. We have modified Table 1 to show the differences between the sample and population across the age groups. We have also acknowledged this sample deviation from the population in the limitations. |
| 4. Table 1 shows that the sample has selection bias introduced because of the sampling methodology used via texts. Women without Internet, younger age group 18 - 24 are less likely to volunteer while the wealthier women are more likely to volunteer. You can address this volunteer bias by standardization if you like or you can just acknowledge it as a limitation. | We have acknowledged this issue further in the limitations section. |
| 5. In table 3, they just presented adjusted utility estimates and p-values without showing unadjusted values. It is not clear how the model building was done. How were the confounders that they adjusted for selected? If the unadjusted values were presented, we can deduce that variables included were indeed confounders. | In the text on page 19, prior to Table 3, the unadjusted values are presented in text. The selection process for the variables in the final LMM are outlined in methods on page 10. We have added some text on page 19 to clarify this. |
| 6. The final model selected in table 3 has a lot of variables that were not statistically significant. Unless they insist on author knowledge as the basis for inclusion irrespective of the evidence from the analysis, we are not sure if some of these variables are just crowding the model. I would suggest they apply Akaike and Bayesian information criteria for the full model and reduced models so that they test model best fit. | The variables in Table 3 were selected as per our methods on page 10: *Predicting variables found to be significantly associated with utility values in initial LMMs were included as fixed effects in a final multivariate LMM. Clinical and demographic variables that were found to be significantly different from the eligible birthing population were additionally included as fixed effects to control for variations in the study sample compared to the birthing population.*  We agree that applying Akaike and Bayesian info criteria as evidence for best model fit would strengthen the results, and have done this in the methods and text immediately prior to Table 3. |
| **Reviewer two comments** |  |
| 1.It is recommended to update the old references (References 4, 24 and 25). | We have updated reference 4 to a contemporary Australian cost analysis.  References 24 and 25 cite the original source for these measurement tools and we think they are the most appropriate references. |
| 2.Be careful in using punctuation marks. In the limitations part of study, (.) is not used at the end of the sentence below: (It should be acknowledged that other Australian maternity services may vary in the quality of care delivered and therefore the outcomes reported in our study may not be generalizable). | Thank you for identifying this error. We have made this change and checked the manuscript for further errors. |
| 3.The discussion of this article can be expanded more. It is recommended to revise and make it more complete. | We have expanded the discussion to include more limitations as per Reviewer 1’ suggestions. |
| 4.It is better to state the explanations of each table exactly before the corresponding table (review the place of inserting the explanations of tables 3 and 4) | We have adjusted the positioning of text so that the explanations of each table are exactly before the corresponding table. |
| **General** |  |
| While revising your submission, please upload your figure files to the Preflight Analysis and Conversion Engine (PACE) digital diagnostic tool, <https://pacev2.apexcovantage.com/>. PACE helps ensure that figures meet PLOS requirements. To use PACE, you must first register as a user. Registration is free. Then, login and navigate to the UPLOAD tab, where you will find detailed instructions on how to use the tool. If you encounter any issues or have any questions when using PACE, please email PLOS at [figures@plos.org](mailto:figures@plos.org). Please note that Supporting Information files do not need this step. | Thank you for this suggestion. We have done this. |
